# Supplementary figures and images for: Meiotic sex chromosome cohesion and autosomal synapsis are supported by Esco2
Source: Life Sci Alliance. 2020 Feb 12;3(3):e201900564. doi: 10.26508/lsa.201900564 (PMC7025286; doi:10.26508/lsa.201900564)

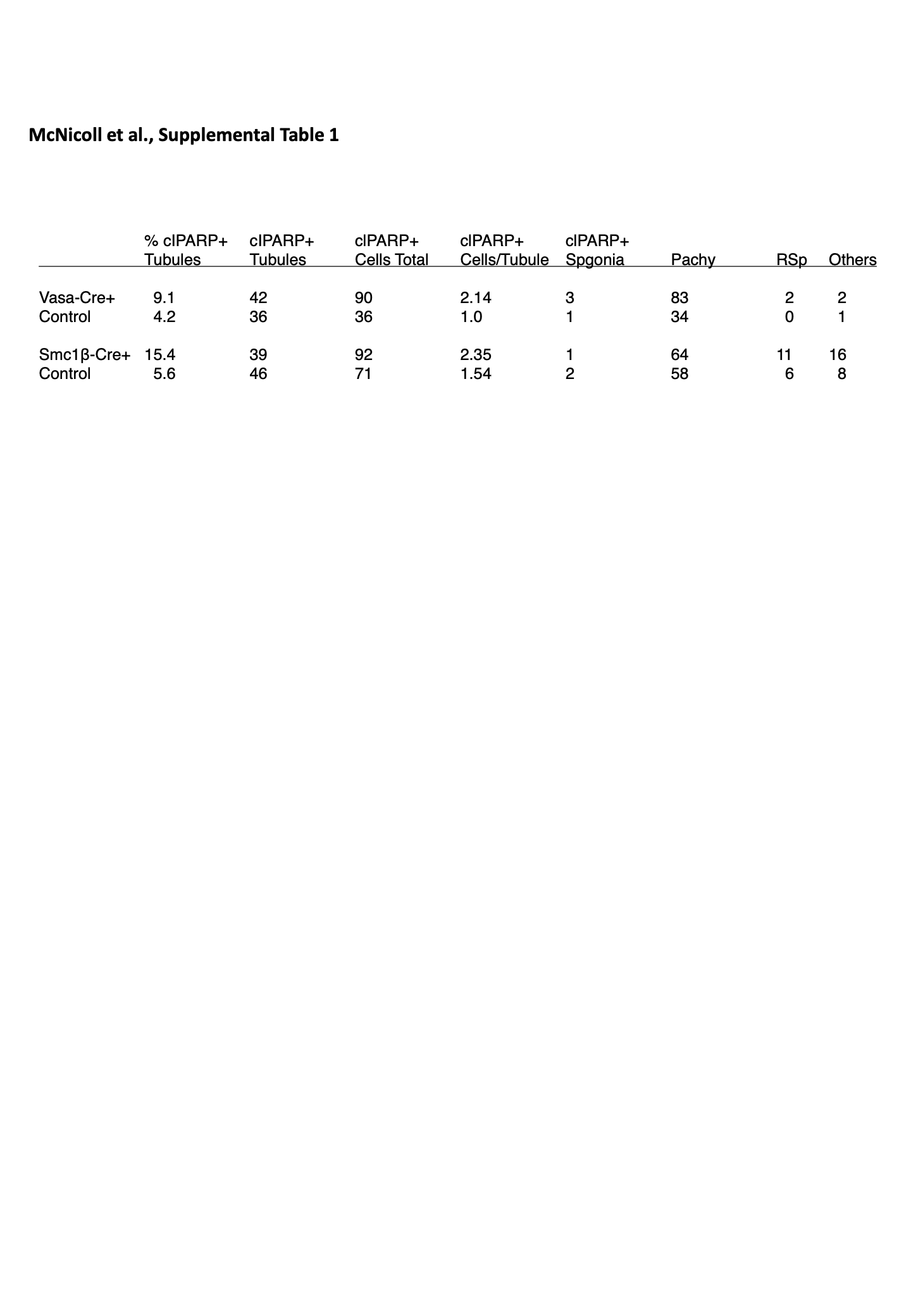

Supplement: Supplementary file 1 [file LSA-2019-00564_TableS1.tif]

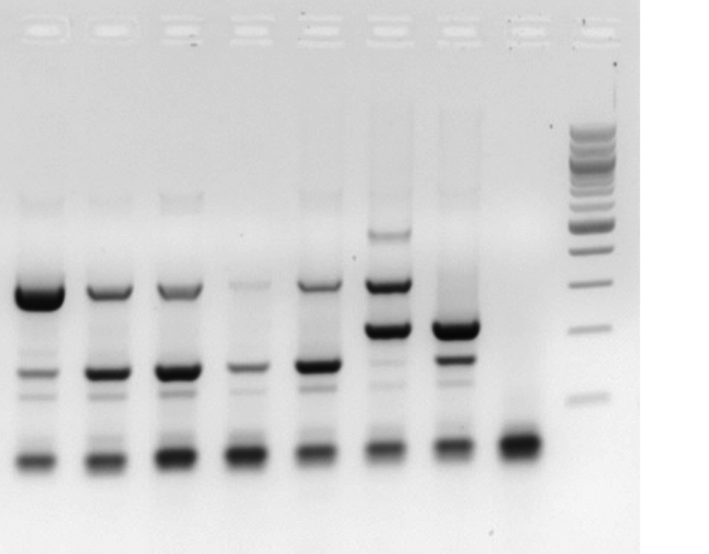

Supplement: Supplementary file 2 [file LSA-2019-00564_SdataFS6.1.tif]

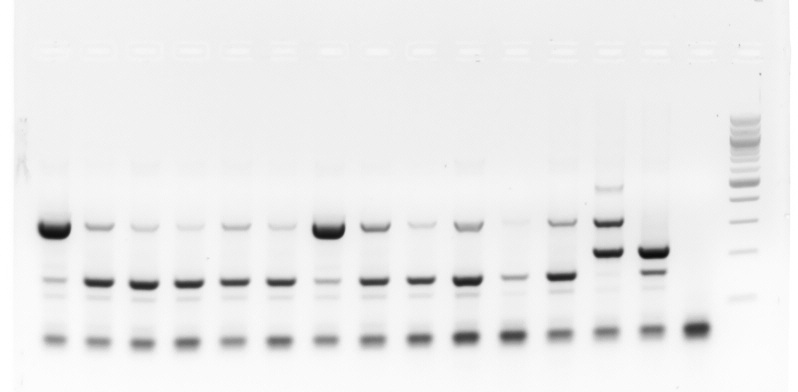

Supplement: Supplementary file 3 [file LSA-2019-00564_SdataFS6.2.tif]

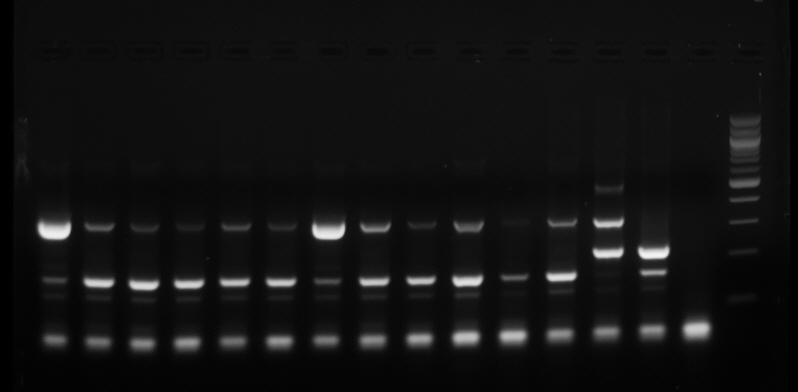

Supplement: Supplementary file 4 [file LSA-2019-00564_SdataFS6.3.tif]
